# Aerobic Exercise Improves the Overall Outcome of Type 2 Diabetes Mellitus Among People With Mental Disorders

**DOI:** 10.1155/da/6651804

**Published:** 2024-12-31

**Authors:** Jiaxuan He, Fan Liu, Peiye Xu, Ting Xu, Haiyang Yu, Baihui Wu, Hanbing Wang, Jia Chen, Kun Zhang, Junbei Zhang, Kaikai Meng, Xiaoqing Yan, Qinsi Yang, Xingxing Zhang, Da Sun, Xia Chen

**Affiliations:** ^1^Institute of Life Sciences and Biomedical Collaborative Innovation Center of Zhejiang Province, Wenzhou University, Wenzhou 325035, China; ^2^Department of Biotechnology, The University of Hong Kong, Hong Kong SAR 999077, China; ^3^Sichuan Provincial Center for Mental Health, Sichuan Provincial People's Hospital, School of Medicine, University of Electronic Science and Technology of China, Chengdu 611100, China; ^4^Chongqing Municipality Clinical Research Center for Endocrinology and Metabolic Diseases, Chongqing University Three Gorges Hospital, Chongqing 404000, China; ^5^Department of Endocrinology, Yiwu Central Hospital, The Affiliated Yiwu Hospital of Wenzhou Medical University, Yiwu 322000, China; ^6^The Chinese-American Research Institute for Diabetic Complications, School of Pharmaceutical Sciences, Wenzhou Medical University, Wenzhou 325035, China; ^7^Wenzhou Institute, University of Chinese Academy of Sciences, Wenzhou 325000, China; ^8^Department of Endocrinology and Metabolism, The First Affiliated Hospital of Wenzhou Medical University, Wenzhou 325000, China

**Keywords:** aerobic exercise, mental disorders, nonpharmacological intervention, pathogenesis, type 2 diabetes mellitus

## Abstract

The escalating global prevalence of type 2 diabetes mellitus (T2DM) and mental disorder (MD) including schizophrenia, bipolar disorder, major depressive disorder, and anxiety highlights the urgency for comprehensive therapeutic strategies. Aerobic exercise (AE) is a viable adjunct therapy, providing significant benefits for individuals dealing with both T2DM and MD. This review consolidates evidence on AE's role in alleviating the physiological and psychological effects of these comorbid conditions. It delves into the pathophysiological connections between T2DM and various MD, including depression, schizophrenia, anxiety, and bipolar disorder—emphasizing their reciprocal exacerbation. Key neurophysiological mechanisms through which AE confers benefits are explored, including neuroinflammation modulation, brain structure and neuroplasticity enhancement, growth factor expression regulation, and hypothalamic–pituitary–adrenal (HPA)/microbiota–gut–brain (MGB) axis normalization. Clinical results indicate that AE significantly improves both metabolic and psychological parameters in patients with T2DM and MD, providing a substantial argument for integrating AE into comprehensive treatment plans. Future research should aim to establish detailed, personalized exercise prescriptions and explore the long-term benefits of AE in this population. This review underscores the potential of AE to complement existing therapeutic modalities and enhance the management of patients with T2DM and MD.

## 1. Introduction

The relationship between type 2 diabetes mellitus (T2DM) and mental disorder (MD), including schizophrenia, bipolar disorder, major depressive disorder, and anxiety disorders, has become a significant area of interest for medical and psychological research. This interest is driven by the high prevalence of comorbidity and the complex bidirectional relationships these conditions share [1, 2]. Globally, the prevalence of T2DM is expected to rise from 10.5% in 2021, affecting 536.6 million individuals, to an estimated 12.2% by 2045, impacting 783.2 million people worldwide [3]. Concurrently, patients with T2DM are particularly susceptible to developing MD, with studies demonstrating a heightened risk ratio even after adjusting for sociodemographic factors [4, 5]. For example, the relative risk of developing schizophrenia, bipolar disorder, and major depressive disorder in diabetic patients is 1.22 (95% CI: 1.06–1.41), 1.58 (95% CI: 1.33–1.87), and 1.59 (95% CI: 1.49–1.70), respectively [6]. These statistics serve to highlight the pressing necessity for the development of comprehensive treatment strategies that integrate both pharmacological and nonpharmacological interventions.

The relationship between T2DM and MD is intricate and complex, with bidirectional influences. Existing literature identifies three pivotal aspects [7]: (I) T2DM and MD frequently co-occur; (II) individuals with pre-existing MD are at a heightened risk of developing T2DM; and (III) those diagnosed with T2DM exhibit an increased vulnerability to MD. The interaction between these conditions is further complicated by physiological challenges and psychological barriers, such as the stigma associated with MD, which can impede timely and appropriate care. This highlights the crucial need for a nuanced understanding and management of their co-occurrence and advocates for a patient-centered approach that seamlessly integrates physical health and mental well-being into more cohesive care strategies [8].

The effective management of T2DM with MD necessitates a holistic strategy (Table 1) that encompasses pharmacological treatments [15], psychotherapeutic interventions [16], and lifestyle modifications [17]. Although pharmacotherapy is fundamental in managing mental health, its effectiveness is often limited by side effects, poor adherence, withdrawal symptoms, and potential misuse [18]. Certain psychotropic medications can exacerbate weight gain and adversely affect diabetes outcomes [19]. Psychotherapeutic methods, while beneficial, demand ongoing commitment and can be emotionally taxing [20]. Consequently, lifestyle interventions, in particular AE, are gaining recognition as a valuable modality for improving both glycemic control and mental health [14]. The therapeutic potential of exercise, particularly in managing mild to moderate mood and anxiety disorders, is increasingly recognized, sometimes rivaling traditional pharmacotherapy and psychotherapy [21, 22]. Integrating exercise into standard treatment plans has been shown to have synergistic benefits [23].

Aerobic exercise (AE), characterized by a moderate intensity and reliance on oxygen, not only improves physical health outcomes, such as cardiovascular health, metabolic rate, and bone density [24], but also offers therapeutic effects for mental well-being [25]. The World Health Organization recommends a minimum of 150 min of moderate-intensity AE per week as a nonpharmacological intervention for managing conditions such as nonalcoholic fatty liver disease, T2DM, and MD [26–33]. Despite the widespread acknowledgment of the benefits of AE for both physical and mental health, the precise mechanisms by which it mitigates psychological challenges in patients with T2DM and MD require further investigation [34, 35].

**Figure 1:**
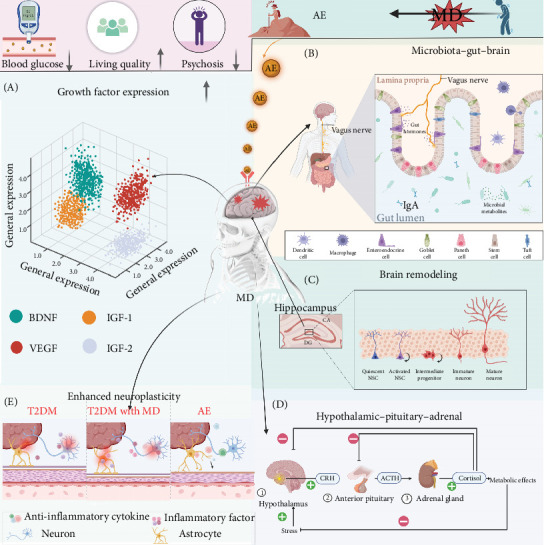
AE as a treatment strategy for T2DM combined with MD. AE can enhance glycemic control, improve quality of life, reduce the occurrence of MD, and also highlight its role in (A) increasing growth factor expression, (B) improving intestinal flora, (C) enhancing brain remodeling, (D) improving hypothalamic–pituitary–adrenal (HPA), and (E) enhancing neuronal remodeling. AE, aerobic exercise; MD, mental disorder; T2DM, type 2 diabetes mellitus.

The objective of this review is to delineate the contributions of AE in the management of T2DM and MD (Figure 1). It will clarify the role of AE within a holistic treatment framework that also includes traditional pharmacotherapy and psychotherapy. The physiological and psychological mechanisms by which AE enhances patient outcomes will be explored, providing a detailed comprehension and guiding future research to refine exercise recommendations for this complex patient group.

## 2. Pathogenic Association and Contributing Factors of T2DM Combined With MD

**Figure 2:**
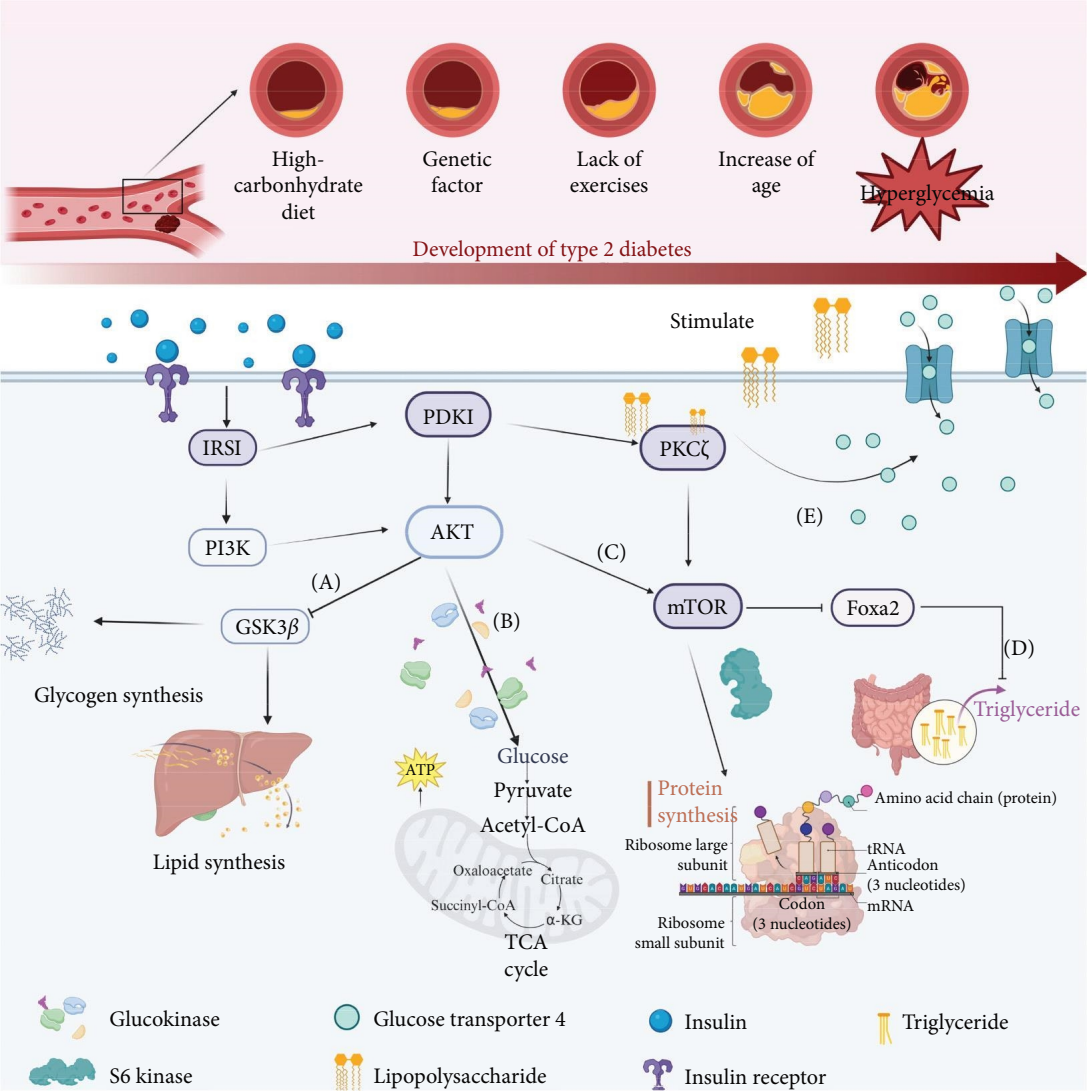
The insulin signaling pathway illustrates the pivotal role of insulin in energy regulation, with the phosphoinositide 3-kinase (PI3K) pathway playing a crucial role. (A) AKT's role in promoting glycogen and fat synthesis by inhibiting glycogen synthase kinase-3 beta (GSK-3β). (B) Direct regulation of glycolysis by AKT through stimulation of glucokinase. (C) Protein synthesis via the mammalian target of rapamycin (mTOR) pathway. (D) Inhibition of triglyceride production by suppressing forkhead box protein A2 (Foxa2). (E) Enhancement of glucose uptake in muscle and fat cells through the phosphoinositide-dependent kinase (PDK) pathway and the translocation of glucose transporter type 4 (GLUT-4) to the cell membrane, also influenced by protein kinase C (PKC) activation.

The pathogenesis of T2DM is the result of a complex interplay of genetic, epigenetic, and environmental factors, which are further compounded by psychological stress and sedentary lifestyles [36, 37]. Central to the pathology of T2DM are insulin resistance and beta-cell dysfunction. Insulin resistance diminishes tissue sensitivity to insulin, impairing glucose uptake, while beta-cell dysfunction leads to reduced insulin production and resultant hyperglycemia [38]. The disease's complexity is further highlighted by the brain's dependence on a consistent glucose supply and the critical role of inflammatory pathways in disease onset (Figure 2). Contributing environmental factors include poor diet, aging, alcohol consumption, smoking, and especially obesity [39], which predisposes individuals to insulin resistance and exacerbates the condition by altering genes critical for insulin signaling and glucose tolerance [40]. In addition, T2DM and MD share multiple biological pathways, and these shared pathways highlight the link between metabolic dysregulation and brain function, showing how changes in glucose levels affect cognitive and mental health [41].

**Figure 3:**
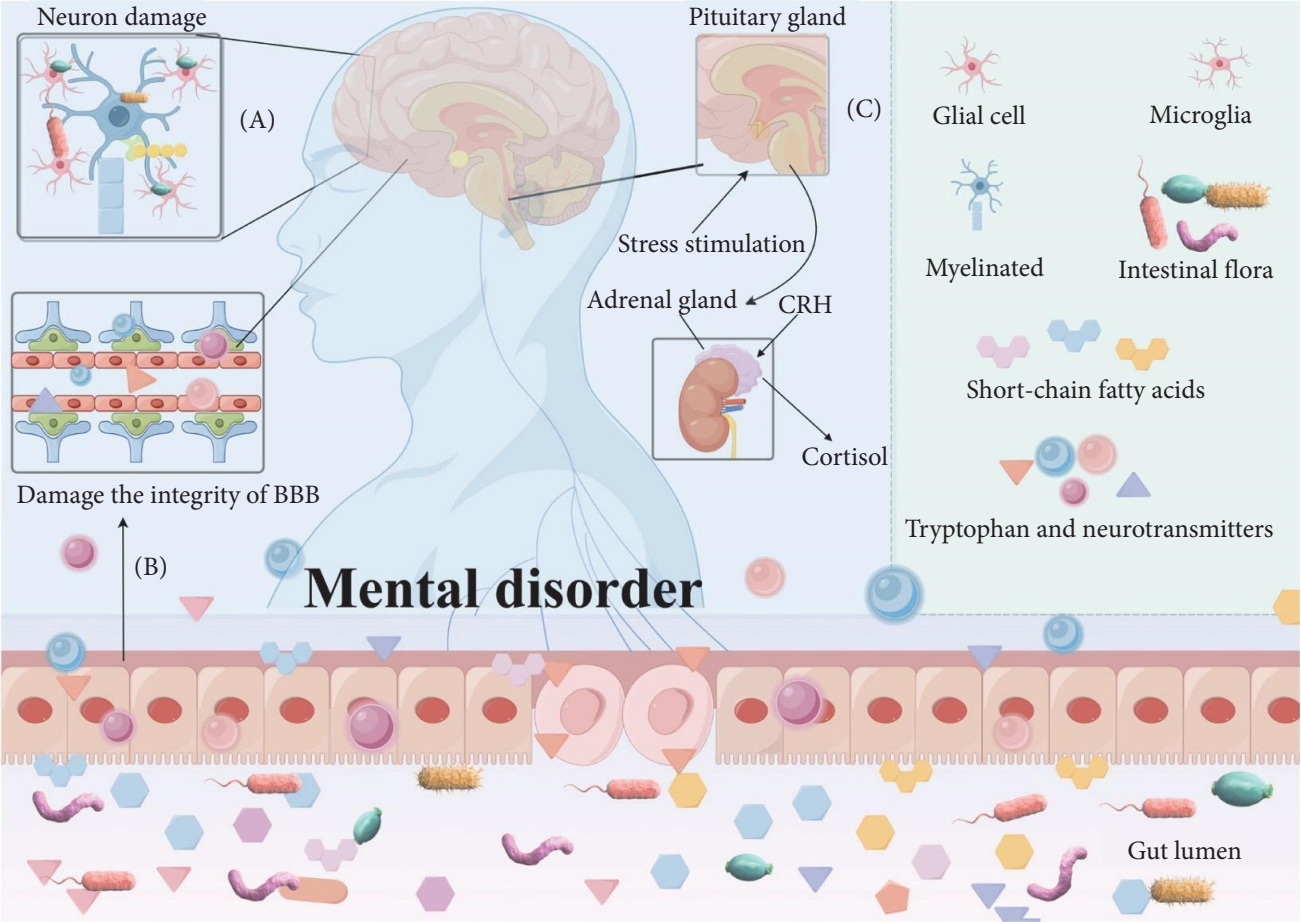
A comprehensive analysis of the MGB/HPA axis in the context of mental health. (A) Direct neuronal damage by gut microbes. (B) Microbial metabolites, including short-chain fatty acids (SCFAs), tryptophan derivatives, cytokines, and neurotransmitters (e.g., serotonin, GABA, and glutamate), can disrupt the integrity of the blood–brain barrier (BBB), affecting brain function and potentially triggering mental disorders. (C) Stress-induced activation of the HPA axis, which initiates the synthesis of corticotrophin-releasing hormone (CRH), results in the release of cortisol and subsequent implications for mental health. GABA, gamma-aminobutyric acid; HPA, hypothalamic–pituitary–adrenal; MGB, microbiota–gut–brain.

The intricate relationship between T2DM and MD is further mediated by the microbiota–gut–brain (MGB) axis, where dysbiosis impacts neurological and mental health through neural, metabolic, and inflammatory pathways [42, 43]. Overactivation of the hypothalamic–pituitary–adrenal (HPA) in MD plays a crucial role in this interplay, influencing glucose metabolism and insulin sensitivity (Figure 3) [44–47]. It is becoming increasingly evident that antipsychotic-induced metabolic disturbances, genetic overlaps, and lifestyle factors also contribute to the comorbidity of MD and T2DM [48–50]. Consequently, a holistic approach is required to gain a deeper understanding of these complex conditions and to develop effective management strategies.

### 2.1. T2DM Combined With Depression

Depression is acknowledged by the World Health Organization as a significant global health concern [51, 52], with a pronounced intersection with T2DM. Epidemiological data indicate a substantial increase in the prevalence of clinically relevant depression among individuals with diabetes [53], far exceeding the rates observed in the general population [54]. From 2007 to 2018, the global prevalence of depression among T2DM patients increased from 20% to 32%. This represents a 36%–64% elevated risk of developing depression among diabetic individuals [55, 56]. The chronic management of diabetes often exacerbates psychological stress, leading to poor metabolic control and a cyclical exacerbation of depressive symptoms. Approximately 80% of T2DM patients with depression experience recurrent depressive episodes within 5 years [57].

A number of shared pathophysiological factors have been identified between T2DM and depression. This includes systemic inflammation, HPA axis dysfunction, insulin resistance, monoaminergic neurotransmitter system dysregulation, chronic stress responses, and gut microbiota dysbiosis [58]. It is proposed that inflammation is a key for depression [59], with approximately one-third of adults with depression exhibiting elevated levels of inflammatory cytokines, including C-reactive protein (CRP), tumor necrosis factor α (TNF-α), and interleukin-6 (IL-6) [60]. These inflammatory markers contribute significantly to both the pathophysiology of diabetes and the behavioral manifestations of depression [61].

The body's response to acute stress involves the activation of the HPA axis, which increases sympathetic nervous system activity, leading to elevated production of adrenaline and noradrenaline [62]. This, in turn, activates the HPA axis and subsequent inflammatory responses, which increase cortisol levels and proinflammatory cytokines. These cytokines contribute to the onset of T2DM with depression and influence insulin resistance and neurobiological changes. Cytokines also impair neurological function by reducing brain-derived neurotrophic factor (BDNF) levels, which in turn leads to a reduction in neurogenesis and synaptic plasticity [63]. Furthermore, they activate indoleamine 2,3-dioxygenase (IDO) in the kynurenine pathway, while cortisol upregulates tryptophan 2,3-dioxygenase (TDO) as rate-limiting enzyme, leading to reduced availability of tryptophan, a crucial precursor for serotonin synthesis [64].

In summary, the relationship between T2DM and depression is characterized by a high prevalence of comorbidity, which is influenced by a complex interplay of biological and psychosocial factors [65]. The bidirectional exacerbation of these conditions is largely driven by the intricate relationship between systemic inflammation and HPA axis dysfunction [66–68].

### 2.2. T2DM Combined With Schizophrenia

Schizophrenia is a SMI that disrupts cognition, emotion, behavior, and the connection between mental activity and the environment [69]. It manifests with positive symptoms such as delusions and hallucinations and negative symptoms including aggressive behavior, which are influenced by both genetic and environmental factors [70]. Schizophrenia has a lifetime prevalence of 1.0%–1.5%, reduces life expectancy by 20%, and doubles the mortality rate compared to the general population [71]. In developed countries, schizophrenia accounts for 5.3%–22% of total national healthcare expenditures [72]. The prevalence of diabetes mellitus in patients with schizophrenia has been reported to be two to three times higher than in the general population, with the prevalence of T2DM in patients with schizophrenia ranging from ~6% to 21% [73]. Studies have shown that schizophrenia combined with diabetes leads to more severe cognitive impairment than schizophrenia alone [74].

The mechanisms underlying the increased prevalence of T2DM in patients with schizophrenia are multifactorial. Both the condition itself and the use of antipsychotics increase the risk of diabetes [75]. Neuronal function and the brain's biochemical processes require a constant glucose supply. However, the central nervous system (CNS) lacks large glycogen reserves and relies heavily on glucose [76]. Alterations in glucose metabolism can severely affect CNS functioning and contribute to schizophrenia. The insulin signaling pathway, involving AKT's modulation of glucokinase, glycogen synthase kinase 3β (GSK3β), forkhead box protein A2 (Foxa2), phosphodiesterase 3B (PDE3B), and glucose transporter type 4 (GLUT-4), plays a central role. In schizophrenia, there is a notable reduction in upstream pS312—insulin signaling protein-1 (IRS-1) signaling and a decrease in downstream serine–threonine kinase pathway activities, including AKT, GSK3β, mTOR, p70S6K [77].

Patients with schizophrenia also exhibit elevated levels of proinflammatory cytokines in their blood and cerebrospinal fluid [78]. These cytokines, upon crossing a compromised blood–brain barrier (BBB), induce neuroinflammation and further disrupt CNS integrity [79]. TNF-α activation of microglia leads to MCP-1 production, which recruits monocytes to the brain, perpetuating inflammation [80]. These inflammatory processes modulate mood and cognitive functions by altering neurotransmitter levels, activating neuroendocrine responses, increasing glutamate (Glu), and impairing brain plasticity [81].

### 2.3. T2DM Combined With Anxiety

Anxiety disorders in patients with T2DM are characterized by persistent, excessive fear and worry in nonthreatening situations [82]. Symptoms commonly include nervousness, panic, fear, and rapid heartbeat [83]. Anxiety symptoms in T2DM patients range from 14% to 40%, with a diagnosed anxiety disorder prevalence of 8.8% to 27.3% [84]. These rates are substantially higher than those observed in the general population, where T2DM patients are more prone to depression and anxiety [85]. The coexistence of T2DM with anxiety frequently results in suboptimal glycemic control, diminished treatment adherence, and a diminished quality of life [86]. Furthermore, anxiety is associated with an elevated risk of cardiovascular disease, including hypertension and an increased incidence of cardiovascular diseases, which can ultimately lead to premature mortality [87].

The bidirectional relationship between T2DM and anxiety is further compounded by the involvement of neurochemical dysregulation [88], immune inflammation [89], HPA axis overactivation [90], and gut microbiota dysbiosis [91]. Central neurotransmitters such as the inhibitory neurotransmitter gamma-aminobutyric acid (GABA), excitatory neurotransmitter Glu, and indoleamine neurotransmitter serotonin (5-HT) play roles in both T2DM and anxiety. GABA, through its GABAA and GABAB receptors, produces anxiolytic effects and has been found to alleviate anxiety symptoms while also modulating immune responses and inducing pancreatic β-cell regeneration [92–94]. Anomalies in GABA synthesis can alter insulin resistance by affecting GLUT-4 levels and gluconeogenesis [95]. Glu, typically elevated in anxiety [96], impacts insulin secretion through Ca^2+^ signaling and can lead to β-cell death by activating N-methyl-D-aspartate receptors [97, 98]. 5-HT, distributed in the central and peripheral nervous systems, exerts antianxiety effects through activation of 5-HT1A, 5-HT2B, 5-HT4, 5-HT6, and 5-HT7 receptors and antagonism of 5-HT2A, 5-HT2C, and 5-HT3 receptors [99]. Research has shown that 5-HT inhibits insulin and glucagon secretion in healthy populations, while overexpression of 5-HT1D and 5-HT2A receptors in T2DM patients significantly increases glucose-stimulated insulin secretion [100], though the exact mechanisms require further investigation.

Elevated serum levels ofinflammatory cytokines, including IL-6, IL-17, and TNF-α, have been identified in T2DM patients with anxiety, mirroring increases observed in animal models [101]. These cytokines reduce the effectiveness of BDNF, impacting its binding to tyrosine kinase receptor B (TrkB) and decreasing insulin sensitivity [102]. The physiologically impacts of anxiety extend to the HPA axis, with effects comparable to those observed in depression.

### 2.4. T2DM Combined With Bipolar Disorder

Bipolar disorder is a severe MD characterized by fluctuations between periods of elevated mood and depression, accompanied by significant physiological, psychological, cognitive, and behavioral disruptions [103]. Globally, ~2% of the population is affected by bipolar disorder [104], with these individuals facing a 52% likelihood of developing insulin resistance or diabetes [105]. The co-occurrence of bipolar disorder and T2DM is associated with a more severe and chronic course, characterized by frequent episodes, increased mental hospitalizations, and a 30% reduction in life expectancy [106].

The neuropsychiatric symptoms of bipolar disorder are influenced by a number of factors, including sleep disorders, mitochondrial dysfunction, and neuroinflammatory responses. These factors also contribute to the development of T2DM. Disrupted sleep, a core symptom of bipolar disorder, activates the sympathetic nervous system activation, reduces insulin secretion, and increases insulin resistance, which collectively result in elevated plasma free fatty acid levels [107, 108]. Mitochondrial dysfunction in bipolar disorder is characterized by decreased aerobic metabolism, reduced mitochondrial membrane potential, and increased mitochondrial DNA deletions [109]. These changes are analogous to those observed in muscle mitochondria in T2DM, which exhibit reduced ATP production and structural abnormalities [110].

It is becoming increasingly evident that immune dysregulation plays a pivotal role in the pathogenesis of both bipolar disorder and T2DM [111]. This is evidenced by the elevated levels of proinflammatory cytokines, such as IL-1Ra, IL-6, and TNF-α, observed in both conditions [112]. These cytokines have been associated with both cognitive decline and diabetes onset. Furthermore, they interact with the HPA axis, affecting glucose metabolism and insulin sensitivity.

Moreover, lifestyle factors commonly observed in bipolar disorder, including physical inactivity, high intake of simple carbohydrates, and side effects from psychiatric medications, exacerbate glucose metabolism issues [113–115]. This necessitates the implementation of a comprehensive management strategy that addresses both the mental and physical health challenges of this patient population.

## 3. Neurophysiological Mechanisms of AE in Improving Diabetes Combined With MD

Pharmacological treatments for diabetes-related MD, while effective in symptom management, have been linked with an increased risk of developing diabetes due to antipsychotic drug use [116]. In contrast, AE has been demonstrated to offer dual benefits for both physical and mental health. Research indicates that women with chronic diseases such as diabetes and depression who engage in vigorous physical activity report markedly reduced depressive symptoms [117]. Similar benefits have been observed in individuals with comorbid diabetes and depression, emphasizing AE's restorative potential [118].

AE is recognized as a safe and straightforward physical activity. Fink et al. [119] report that AE can significantly promote blood circulation, increase brain oxygen supply, stimulate the production of neurotrophic factors, and promote the establishment of neural synapses. These changes reduce cortical atrophy, enhance the integrity of the cerebral cortex, improve cognitive function in elderly patients with diabetes, and slow the progression of cognitive impairment.

Regular engagement in AE serves as a protective factor against diabetes and MD [120]. High-intensity physical activities are particularly effective in guarding against diabetes, obesity, and hypertension [121]. A meta-analysis involving 42,264 patients revealed that exercise markedly improves anxiety levels [122]. The association between physical inactivity and an increased risk of major noncommunicable diseases highlights the importance of AE in clinical management strategies that combine regular exercise with medication for enhanced health outcomes [123].

### 3.1. The Impact of AE on Neuroinflammation

Insulin resistance has been demonstrated to impact brain structure and function by activating inflammatory pathways. Furthermore, hyperglycemia-induced neuroinflammation has been shown to disrupt the BBB, facilitating the infiltration of inflammatory mediators into the brain, which in turn exacerbates neuronal damage [124]. The activation of microglia, a key event in T2DM [125], has been shown to promote inflammation and neural injury, impacting mood regulation and cognitive functions in disorders such as depression and bipolar disorder [126, 127], and contributing to symptoms such as hallucinations in schizophrenia and characteristic symptoms in anxiety disorders [128–130]. Neuroinflammation in anxiety disorders, especially in stress-related autism spectrum disorders, affects brain development and function, leading to characteristic symptoms [131, 132].

AE induces muscle contraction that trigger the release of anti-inflammatory cytokines, also known as myokines, such as IL-6, IL-10, and IL-1 receptor antagonist [133]. These myokines mediate health benefits by creating an anti-inflammatory environment and reducing proinflammatory proteins, such as TNF-α and CRP, which are linked to insulin resistance and hyperglycemia [134–136].

It has been demonstrated that exercise-induced reduction in myostatin results in decreased muscle fat and enhanced glucose metabolism [137]. Studies have indicated that moderate to high-intensity aerobic or resistance exercise increases IL-6 (145%) and neutrophil count (51%) [138] and has been shown to improve cognitive impairments in T2DM by modulating pathways such as AMPK/SIRT1 and reducing JAK2/STAT3 signaling [139]. Further research indicates that AE reduces brain inflammation and improves metabolic and cognitive markers in diabetic conditions [140].

**Figure 4:**
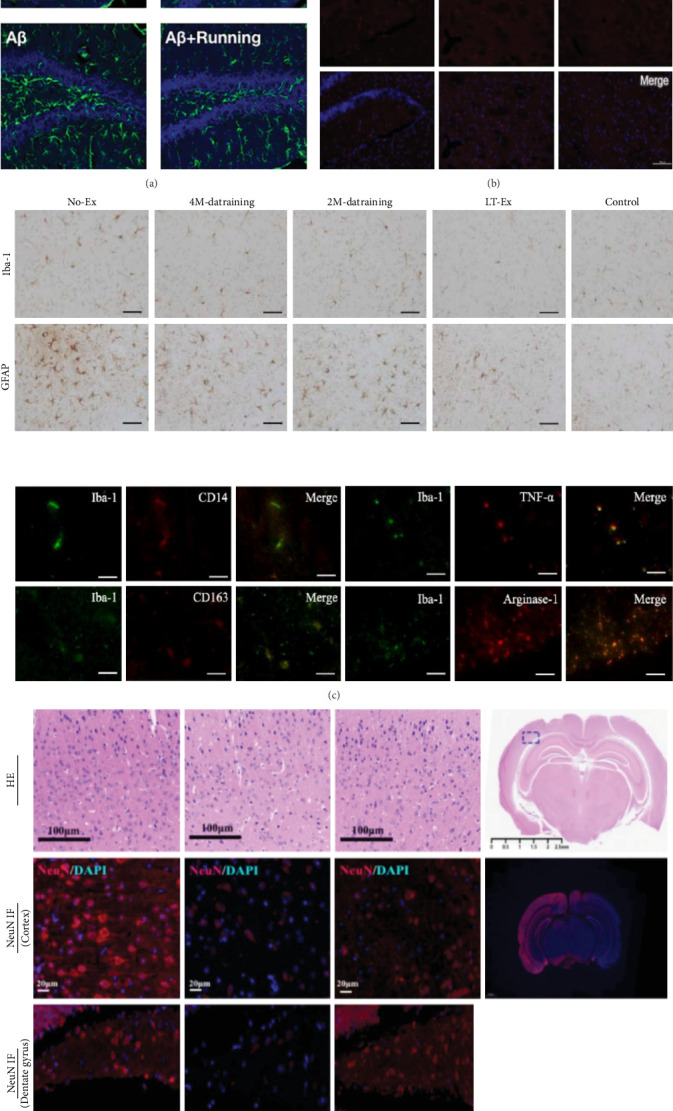
Neuroinflammatory responses to aerobic exercise (AE). (A) Confocal immunofluorescence showed a lower density of GMAP-labeled astrocytes in the dentate gyrus region that received treadmill exercise in the Aβ treatment group compared to the nonexercise control group [141]. Copyright 2018 Elsevier. (B) Immunohistochemical results showed that AE decreased the activation level of M1 in the hippocampus of diabetic mice [142]. Copyright 2020 Elsevier. (C) AE reduces neuroinflammation in the brain [143]. Copyright 2023 Elsevier. (D) Treadmill exercise remarkably decreased the cortical interstitial space, increased the number of NeuN (+) neurons in the cortex and dentate gyrus [144]. Copyright 2024 Springer.

The basic research on the effects of exercise on neuroinflammation is shown in Figure 4.

### 3.2. The Impact of AE on Brain Structure Changes and Neural Plasticity

**Figure 5:**
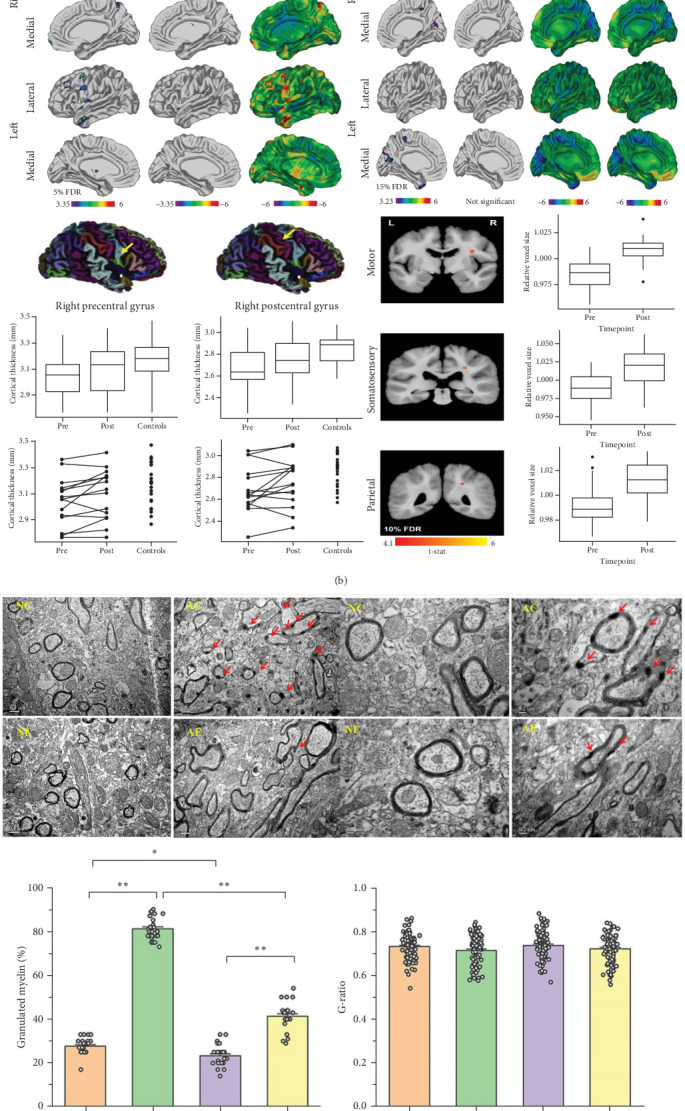
The study on aerobic exercise's (AE's) impact on brain structural changes and neuroplasticity. (A) In schizophrenia patients, AE was associated with significantly changes in hippocampal volume, particularly affecting the oligodendrocyte precursor gene burden and the radial glia-like polygenic score, suggesting genetic influences on brain volume response to exercise [145]. Copyright 2019 Nature Portfolio. (B) Compared with healthy controls, cortical thickness in key brain regions increased after AE, highlighting the structural benefits of exercise on brain morphology [146]. Copyright 2018 Elsevier. (C) AE mitigated structural disturbances in the myelin sheath within the temporal lobe of Alzheimer's disease models, showcasing its potential in addressing neuropathological changes [147]. Copyright 2023 Elsevier. *⁣*^*∗*^*p* < 0.05, *⁣*^*∗∗*^*p* < 0.01.

As shown in Figure 5, AE has been demonstrated to significantly enhance brain function, cognition, and memory while improving cellular redox states. Besides, AE has been shown to increase hippocampal volume and promote neurogenesis and synaptic genesis, which are crucial for maintaining cognitive functions and delaying cognitive decline [148]. Additionally, AE fosters brain plasticity and adaptive behavioral changes, reducing depressive symptoms and enhancing cognitive control [149].

The role of AE in neurogenesis is particularly evident in the hippocampus, an area essential for learning processes dependent on hippocampal function [150]. Animal studies have demonstrated the effectiveness of AE in stimulating neurogenesis within the hippocampal dentate gyrus. Recent research suggests that AE could be a potential strategy for boosting hippocampal neurogenesis in adults [151], with implications for diseases like multiple sclerosis, where AE enhances both functional and structural connectivity across critical brain regions [152]. These neuroadaptive processes reflect the brain's capacity to reorganize and improve its health, thereby reinforcing AE's neuroprotective attributes.

These insights suggest that AE has the ability to induce functional changes in brain regions associated with movement, promote neuroplasticity, and potentially reverse regional brain matter loss. It is a new possibility to use AE as a therapeutic intervention to promote brain health and mitigate aging and neurodegenerative diseases.

### 3.3. AE Regulation of Growth Factor Expression

Recent studies have highlighted the pivotal role of AE in modulating neurotrophic factors, thereby facilitating neuroplasticity [153]. BDNF, a key player in this process, not only stimulates neurogenesis but also modulates the hypothalamic–pituitary axis to reduce cortisol levels, which are crucial for mitigating depressive behaviors. The widespread presence of BDNF across the the CNS, particularly in regions such as the hippocampus and amygdala, and its interaction with the Trk-B receptor, activates signaling pathways (PI3K and ERK1/2) that are essential for synaptic transmission and neurogenesis [154].

**Figure 6:**
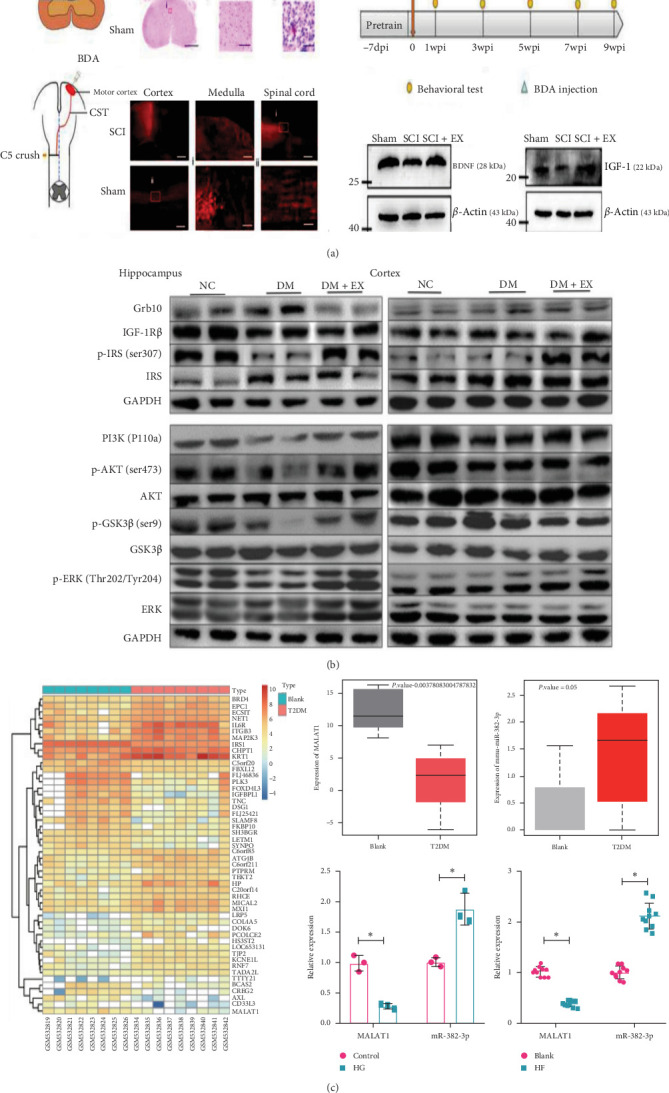
Aerobic exercise's (AE's) regulation of growth factor expression. (A) Treadmill training postspinal cord injury significantly upregulated BDNF and IGF-1 expressions [158]. Copyright 2023 Springer. (B) In diabetic rats, treadmill training modulated several key metabolic pathways, enhancing insulin signaling and reducing inflammatory markers [159]. Copyright 2022 Elsevier. (C) AE increased MALAT1 expression in serum exosomes of T2DM mice [160]. Copyright 2023 BMC. BDNF, brain-derived neurotrophic factor; T2DM, type 2 diabetes mellitus.

A significant elevation in peripheral BDNF levels following AE has been demonstrated to correlate strongly with cognitive improvements [155]. Meanwhile, a study on female Wistar rats demonstrated that AE led to an increased BDNF expression, particularly in those without ovaries [156], suggesting a potential link between exercise, estrogen levels, and neurotrophic expression. Furthermore, AE has been demonstrated to elevate levels of other growth factors, including insulin-like growth factor (IGF-1), vascular endothelial growth factor A (VEGFA), and fibroblast growth factor 2 (FGF-2), in the hippocampus by 92%, 37%, and 43%, respectively [157]. These findings highlight that AE can influence brain function by modulating the expression of these growth factors (Figure 6).

Besides BDNF, IGF-1 and VEGF are integral to the chronic effects of AE on neurovascular and neurocognitive health. These factors, enhanced by AE, promote endothelial growth and cross the BBB to improve synaptic plasticity and neuronal survival. VEGF secretion is notably increased by skeletal muscle activity during long-term exercise, further underscoring the systemic benefits of AE [161]. The collective findings demonstrate the significant impact of AE on the enhancement of brain function, which is achieved through the regulation of neurotrophic factor expression, the fostering of neuronal maturation, proliferation, and survival. These processes are essential for cognitive function and overall neurological health.

### 3.4. HPA Axis and MGB Axis Interaction

In MD, there are significant changes in the composition of the gut microbiota, characterized by decreased levels of beneficial bacteria such as *Lactobacillus*, *Bifidobacterium*, and *Faecalibacterium* and increased levels of potentially harmful bacteria such as *Eggerthella*. These changes affect the metabolism of neurotransmitters such as Glu and GABA, thus affecting mental health outcomes.

The gut microbiota interacts with the brain through direct pathways, such as the vagus nerve, and indirect pathways, including metabolites such as short-chain fatty acids (SCFAs), cytokines, and neurotransmitters such as serotonin and GABA. These interactions can alter the integrity of the BBB, affecting brain function and potentially exacerbating MD [162]. Additionally, the gut microbiota regulates histone modifications in various gut immune cells and controls gut inflammation through DNA methylation programming [163, 164].

AE can increase human and mouse microbiome diversity and functional metabolism, affecting the gut and reversing diseases related to obesity, metabolic disorders, poor diet, and neurological and behavioral disorders [165]. AE affects the gastrointestinal tract and shortens the one-time excretion time, thus reducing the contact time between pathogens and the mucosal layer of the gastrointestinal tract [166]. Even in the presence of a high-fat diet, exercise can reduce inflammatory infiltration and protect the morphology and integrity of the gut [167].

Changes in the gut microbiome can affect gastrointestinal functions (secretion, motility, and integrity) and higher brain functions (e.g., neurotransmission, neurogenesis, and behavior) [168], with these effects being bidirectional. Exercise has been shown to improve symptoms of irritable bowel syndrome, stabilize tight junction protein barriers, and is associated with reduced psychological disorders (e.g., depression and anxiety), promoting neurogenesis (through BDNF) and improving HPA axis control [148]. The extent to which exercise's effects on the gut and brain are mediated by changes in the microbiome is significant. AE has been shown to lead to increased microbiome diversity and an increase in Firmicutes phyla producing SCFAs; additionally, athletes exhibit higher levels of the *Akkermansia* genus, associated with metabolic and neurological diseases [165].

On the other hand, the HPA axis plays a critical role in the stress response and is often dysregulated in MD [149], exacerbating metabolic disorders such as diabetes [169]. The gut microbiota influences the HPA axis through microbial antigens, cytokines, and prostaglandins, with stressors such as maternal separation affecting microbial composition [170–172]. Butyrate, a byproduct of beneficial bacteria, is essential for maintaining mucosal integrity and reducing inflammation, which is critical for both mental and gastrointestinal health [173–175]. Epigenetic regulation by butyrate plays a central role in modulating the HPA axis, affecting the levels of BDNF and other stress-related mechanisms [176–178].

**Figure 7:**
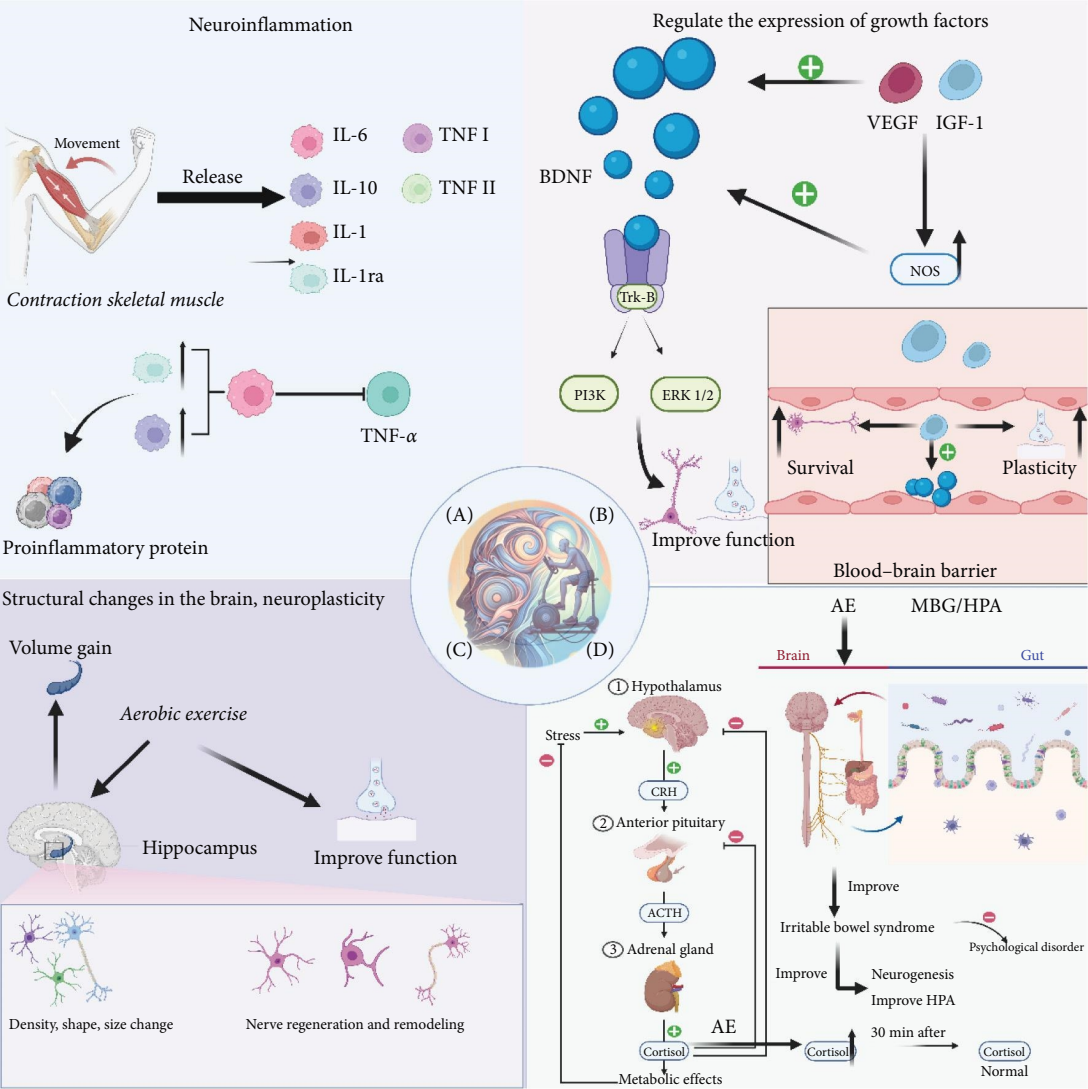
(A) Aerobic exercise (AE) stimulates the synthesis of anti-inflammatory “muscle factors” such as IL-6, IL-10, and IL-1ra through skeletal muscle contraction while reducing the activity of proinflammatory factors such as TNF-α. (B) BDNF binds to the receptor Trk-B and activates the PI3K and ERK1/2 signaling pathways, affecting neurogenesis and synaptic transmission. BDNF interacts with IGF-1 and VEGF, both of which can stimulate the growth of endothelial cells and express NO synthase. Exercise is associated with the increase of peripheral IGF-1 levels. IGF-1 crosses the BBB, enhances synaptic plasticity and neuronal survival, and increases BDNF concentrations. (C) AE enhances neuroplasticity and neurogenesis by improving brain cognition, increasing hippocampal volume and synaptogenesis. (D) Exercise regulates intestinal flora, improves HPA axis function, reduces psychological disorders, promotes neurogenesis, and increases cortisol concentration but quickly returns to normal after exercise and benefits the health of the hypothalamic axis. BBB, blood–brain barrier; BDNF, brain-derived neurotrophic factor; IGF-1, insulin-like growth factor; VEGF, vascular endothelial growth factor.

AE increases cortisol concentrations during the activity but quickly reduces them to normal levels postexercise. Regular moderate-intensity AE of over 30 min is a rehearsal for the stress system, promoting healthy functioning of the HPA axis [179]. Therefore, AE plays a critical role in the regulation of both the HPA and MGB axes, highlighting its importance in integrated care approaches for the management of diabetes combined with MD (Figure 7).

## 4. AE Intervention in T2DM Combined With MD

The World Health Organization has identified insufficient physical activity as a significant global health risk, with inactive associated with a 20%–30% increased risk of mortality compared to active individuals [180]. This lack of exercise is strongly correlated with enhanced morbidity and mortality rates from T2DM, a condition characterized by reduced insulin sensitivity, diminished metabolism, and poor glucose utilization [181]. Physical activity significantly mitigates these risks by improving cardiorespiratory fitness and metabolic indices such as fasting blood glucose, triglycerides, and cholesterol levels [182]. Furthermore, the Lancet Psychiatry Commission has identified lifestyle factors, particularly sedentary behavior and poor diet, as significant contributors to the disease burden in patients with MD, including schizophrenia and bipolar disorder [183]. Regular exercise is recommended to mitigate these risks, underscoring the dual benefits of physical activity in managing both T2DM and mental health conditions.

The integration of AE into treatment protocols is of paramount importance, as it plays a multifaceted role in improving blood sugar control and cardiovascular health, and significantly reducing symptoms associated with MD. Exercise not only addresses physical health but also enhances mental well-being, increasing self-efficacy and quality of life, which are essential for the long-term management of these chronic conditions. This underscores the necessity of a multidisciplinary approach in the treatment of T2DM combined with MD, where AE serves as a cornerstone in holistic patient care.

### 4.1. Basic Research and Clinical Practice in Managing T2DM and Schizophrenia

Patients with T2DM and schizophrenia must navigate complex challenges that demand holistic management approaches. T2DM necessitates strict glucose control via diet, meds, and lifestyle changes, while schizophrenia's effects on cognition and emotion can impede treatment adherence. Symptoms like social withdrawal lead to inactivity, and antipsychotics may foster weight gain and metabolic issues, increasing diabetes risks. Psychiatric decline can significantly hinder diabetes management efficacy [184]. Sustained interventions that combine diabetes self-management education with tailored lifestyle changes are critical, particularly for improving adherence in patients with schizophrenia [14]. Menza et al. [185] conducted a 12-month intervention that integrated diet and AE specifically for patients with schizophrenia and schizoaffective disorders, resulting in significant improvements in weight, body mass index (BMI), nutrition knowledge, and glycemic control, thereby reducing T2DM risk.

AE modalities such as tai chi and aerobic dance are beneficial adjunctive therapies for schizophrenia. Structured programs such as an 8-week supervised aerobic dance program and a 12-week tai chi program have shown substantial improvements in weight, BMI, motor function, and mental health outcomes, underscoring the potential of AE in rehabilitation settings [186–188]. In addition, a 6-month AE walking program (participants walked 5 days a week, 1 h per day, gradually increasing distance and pace to 4 km per day, maintaining this routine until the sixth month) demonstrated notable clinical and metabolic improvements, including better management of metabolic syndrome and improved cardiovascular and metabolic markers [189].

AE has been demonstrated to enhance glycemic control, insulin sensitivity, mental health, and manage symptoms associated with schizophrenia [190]. Therefore, it is essential to implement personalized exercise programs tailored to individual patient conditions. And continuous monitoring by healthcare teams is crucial for optimizing treatment strategies [191].

### 4.2. Basic Research and Clinical Practice of AE in T2DM Patients With Bipolar Disorder

Bipolar disorder is notably linked with elevated risk of cardiovascular diseases and T2DM. Research indicates a higher incidence of cardiovascular risk factors such as dyslipidemia, metabolic syndrome (MetS), and T2DM among bipolar disorder patients [192–194]. Lifestyle factors, including poor diet, inadequate physical activity, and suboptimal sleep, contribute significantly to these comorbidities. Consequently, lifestyle modifications, including dietary changes, increased physical activity, and weight management, are crucial for reducing cardiovascular risks and promoting overall health and well-being [195]. Furthermore, these changes can positively impact mental health and enhance adherence to treatment, thereby improving both metabolic and psychiatric outcomes.

Physical activity, particularly AE, has been demonstrated to support cognitive functions by promoting neurogenesis in the hippocampus, a region essential for memory. Experimental evidence indicates that AE improves blood perfusion to the hippocampus, potentially enhancing memory capabilities. Neurotrophic factors, such as BDNF, IGF-1, and others, are elevated following AE, crossing the BBB and contributing to neuroplasticity and cognitive improvements [196–198]. These findings underscore AE's role in improving the neural architecture and cognitive functions in bipolar disorder, with BDNF playing a key role in synaptic formation and cognitive enhancement [199–201].

In clinical settings, a study by Schuch et al. [202] demonstrated that a single session of maximal AE (participants underwent a maximal aerobic test on a bicycle ergometer, starting with a 5-min warm-up, initial load of 75 W for men and 50 W for women, increasing by 25 W every 60 s until test completion) resulted in significantly higher serum BDNF levels in bipolar disorder participants compared to healthy controls. Furthermore, a 24-week AE walking program involving bipolar disorder patients demonstrated reductions in depression, anxiety, and stress levels, emphasizing the mental health benefits of regular AE [203].

### 4.3. Basic Research and Clinical Practice of AE in T2DM Patients With Depression

Depression is frequently associated with reduced physical activity, and there is a significant correlation between depression and MetS, as well as an elevated risk of T2DM and cardiovascular diseases [204, 205].

AE has been demonstrated to improve cardiopulmonary health and mitigate MetS factors effectively. In a study by Kerling et al. [206], patients who engaged in an enhanced exercise regimen, which included three 45-min aerobic sessions per week, demonstrated notable improvements in exercise capacity, cardiorespiratory fitness, waist circumference, high-density lipoprotein (HDL) cholesterol levels, and a significant reduction in depression scores as measured by the Montgomery Depression Rating Scale (MADRS). Furthermore, de Groot et al. [207] evaluated the combined impact of an exercise regimen and cognitive behavioral therapy (CBT) on adults with T2DM and depression, noting substantial improvements in both depressive symptoms and glycemic control. Similarly, a 6-month randomized study by Schneider et al. [208] on women with diabetes and depression demonstrated that vigorous exercise significantly lessened the severity of depressive symptoms, supporting the therapeutic potential of AE in this demographic. And a meta-analysis observed similar beneficial effects in patients with combined diabetes and depression [209].

The comprehensive benefits of AE extend beyond depression alleviation to enhancing self-esteem, reducing anxiety, and improving sleep, as evidenced by the research of Gilani and Feizabad [210]. de Groot et al. [211] further substantiate the efficacy of combining exercise with CBT in managing severe depression among diverse populations with T2DM. Notably, AE was also found to surpass basic body awareness therapy (BBAT) in alleviating depressive symptoms and improving cardiovascular health [212].

Clinical practice advocates for the integration of AE into holistic treatment plans for depression in patients with T2DM [207, 213]. This approach involves the creation of personalized exercise schedules, the frequent monitoring of progress, and the potential coupling of exercise with psychotherapeutic strategies like CBT in order to optimize treatment outcomes. This integrated treatment model aims to enhance therapeutic efficacy, decrease reliance on medications, bolster self-management in patients, and concurrently mitigate the risk of cardiovascular complications.

### 4.4. Basic Research and Clinical Practice of AE in T2DM Patients With Anxiety Disorder

Anxiety disorders frequently coexist with T2DM, significantly complicating management and treatment outcomes. Smith et al. reported that ~50% of T2DM patients could be diagnosed with generalized anxiety disorder, a rate significantly higher than in nondiabetic populations, with a 20% increased prevalence of anxiety disorders among diabetics [86, 214]. AE has demonstrated efficacy in reducing both diabetic symptoms and anxiety manifestations.

Animal models have provided valuable insights into the physiological mechanisms by which AE mitigates these conditions. For instance, swimming exercise in T2DM C57BL/6 mice notably decreased insulin resistance and brain oxidative stress, thereby alleviating anxiety-like behaviors [215]. Similarly, moderate-intensity treadmill exercise for 5 weeks showed anxiolytic effects in both diabetic and nondiabetic rats [216].

These findings were corroborated by clinical studies. Luo et al. [217] observed significant improvements in HbA1c levels and reductions in symptoms of depression and anxiety in T2DM patients participating in eight trigrams boxing, a form of traditional Chinese exercise. Abdelbasset et al. [218] reported that proprioceptive exercise training led to significant enhancements in functional capabilities and psychological states in diabetic patients suffering from neuropathy. Furthermore, a 12-week moderate-intensity treadmill program significantly improved anxiety, depression, and social well-being in sedentary women with T2DM, underscoring the broad efficacy of AE in managing these symptoms [219]. Beyond that, yoga was effective in improving anxiety in T2DM patients [220].

The incorporation of AE into treatment regimens for T2DM patients with anxiety disorders not only addresses physical symptoms but also enhances mental health outcomes. Such interventions require a comprehensive approach, integrating psychological therapies like CBT and substantial social support to improve patient education, treatment adherence, and overall quality of life [221]. The efficacy of these interventions is contingent upon the implementation of individualized exercise regimens that are tailored to the specific needs and conditions of each patient. This underscores the significance of professional guidance and regular follow-ups. Basic and clinical studies on the intervention of AE in T2DM with MD are summarized in Table 2.

## 5. Challenges and Future Directions

**Figure 8:**
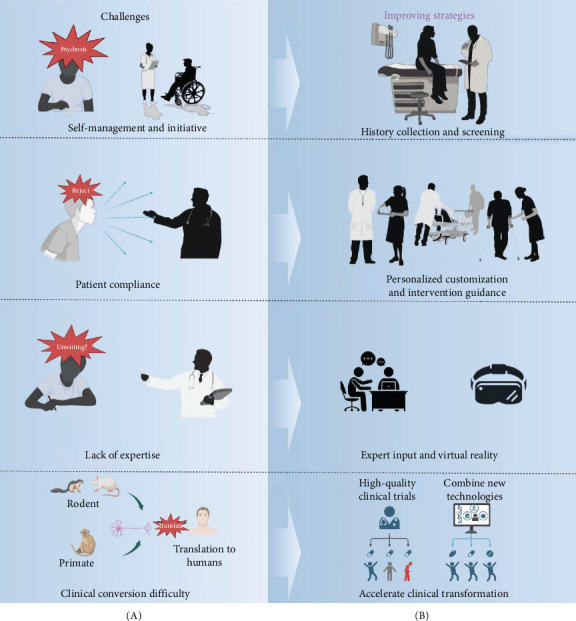
Challenges of aerobic exercise (AE) in the treatment of type 2 diabetes mellitus (T2DM) with mental disorder (MD) (A) and corresponding improvement strategies (B).

As the understanding of the relationship between T2DM and MD advances, it becomes increasingly evident that AE can play a transformative role in managing these co-occurring disorders. However, the integration of AE into treatment plans is not without challenges. The following sections outline the difficulties encountered in implementing AE, the critical role of tailored interventions, and the promising future directions for research and clinical practice (Figure 8).

Patients with diabetes complicated by MD often encounter difficulties with treatment compliance and self-management due to their mental state. Complications related to diabetes can impair physical activity, dampening exercise motivation. The abstract nature of AE intensity guidelines can lead to low patient engagement in daily exercise routines [231]. The stigma associated with mental illness may deter patients from seeking necessary help, complicating the assessment of their condition and the development of effective treatment plans. Therefore, meticulous medical history collection and screening are critical for diagnosing subclinical depressive states.

Interdisciplinary teamwork is essential for deepening understanding of these conditions and integrating diverse treatment plans effectively. Moreover, understanding the motivation behind patients' exercise preferences is crucial for long-term engagement in physical activities. High-intensity exercises may reduce compliance [232], underscoring the necessity for tailored exercise interventions that may include expert input or innovative methods such as virtual reality (VR) [233] to enhance engagement and effectiveness.

Self-management and personalized exercise strategies are of paramount importance due to the unique nature of mental conditions and the general lack of professional knowledge among patients. Methods to enhance compliance include the following: (1) doctor–patient communication is essential for building trust and understanding, which in turn improves diagnosis clarity and compliance. (2) Providing accurate information about medical histories and treatment plans is also crucial for enhancing compliance. (3) The complexity and gradual nature of behavior change are acknowledged, and the use of the model of practice and the health belief model to influence behavior is recognized. The development of a health education framework that addresses exercise barriers and promotes a healthy lifestyle is a crucial aspect of this process [234].

While animal studies have shown promising neurobiological mechanisms, translating these findings to human applications remains challenging. More detailed human studies are needed, focusing on individual responses and incorporating new technologies to enhance research depth and applicability. Furthermore, well-designed high-quality multicenter clinical trials are recommended to evaluate the efficacy of AE in managing T2DM with MD.

## 6. Conclusion

AE constitutes a pivotal component in the management of T2DM and MD, offering substantial improvements in both physiological and psychological health outcomes. The customization of exercise protocols significantly enhances their effectiveness, underscoring the importance of tailored therapeutic approaches. Future research endeavors should concentrate on delineating the specific effects of AE on various MDs and cognitive functions over time, enhancing our understanding of its benefits across different patient demographics. The integration of AE into clinical practice guidelines and the validation of its therapeutic impacts through methodologically sound studies are critical for enhancing the treatment paradigms for T2DM and MD. Such advancements not only promise to improve patient care but also offer promising new avenues for research and development in mental health treatment strategies.

## Figures and Tables

**Table 1:** Advantages and disadvantages of different intervention methods.

Means of intervention		Mechanism	Advantage	Disadvantage	References
Drug intervention therapy	Diabetes drugs (e.g., metformin, pioglitazone)	Medications help regulate carbohydrate and fat metabolism by increasing insulin sensitivity and reducing the amount of glucose produced and released by the liver. Weight loss drugs or antiobesity drugs usually act on the gastrointestinal tract by reducing the absorption of dietary fat, stimulating energy expenditure and reducing fat storage or reducing appetite. Diabetes combination drugs allow patients to switch between treatments based on clinical response	The effect of the drug on the disease is fast, and the patient's compliance is high	Gastrointestinal irritation, long-term use can lead to drug dependence. Some antipsychotics have metabolic side effects that can lead to weight gain and promote the onset of T2DM	[9, 10]
	Weight loss drugs (e.g., amantadine, orlistat, sibutramine)				
	Combinations of weight loss and diabetes drugs (e.g., amantadine with metformin and zonisamide; metformin with amantadine and zonisamide; metformin and sibutramine)				

Psychological intervention therapy	Cognitive behavioral therapy (CBT)	Help people understand and master their emotions and behaviors, enhance self-cognition and self-control ability, and prevent and alleviate the occurrence of psychological problems	The recurrence rate is lower than that of drug therapy, which has more personalized characteristics	The patient needs to self-manage, the treatment time is long, and the treatment cost is high. It has no direct effect on T2DM	[11, 12]
	Psychological intervention				

Lifestyle intervention	Diet	Develop a healthy lifestyle and eliminate behaviors and habits that are detrimental to your physical and mental health	Focusing on changing unhealthy lifestyles has long-term stable effects	Patients have low self-care compliance and need to adhere to it for a long time	[13, 14]
	Motion				
	Diet + exercise				
	Diabetes knowledge education + diet + exercise				

Abbreviation: T2DM, type 2 diabetes mellitus.

**Table 2:** Basic and clinical study of AE intervention in T2DM with MD.

Intervention of AE in T2DM with MD		Study design and diagnosis	Sample	Intervening measure	Intervention time	Result	Reference
Schizophrenia	Clinical research	Randomized controlled trial	60	The experimental group received the aerobic dance program intervention for 8 weeks (60 min, twice a week, intensity: 60%–79% of predicted HRmax, supervised by a coach), and the control group received no intervention. Participants were evaluated based on outcome variables such as body weight, body mass index (BMI), muscular endurance, flexibility, and cardiorespiratory endurance. Variables were measured before intervention (pretest), after intervention (post-test), and 12 weeks after intervention (follow-up)	8 weeks	In both testing and follow-up, the experimental group's body weight and BMI were reduced, and significant differences were seen between groups in all health-related fitness outcomes except muscle endurance	[186]
		Randomized controlled trial	153	(A) Tai chi: 22 simple movements (B) Moderate AE to achieve 50%–60% of maximal oxygen consumption	12 weeks	Compared with CG, motor deficits were significantly reduced, and posterior finger spread and mean cortisol increased, motor deficits, negative, and depressive symptoms were significantly reduced in the exercise group, and forward finger spread, function of daily living, and mean cortisol increased in the exercise group	[187]
		Randomized controlled trial	244	Course 2 times/month, 120 min: 45 min social skills training, 45 min tai chi, 30 min rest Supervised by three full-time psychiatrists and an assistant	12 months	Compared with the medication alone group, the combined intervention group had lower scores on the Positive and Negative Symptom Scale (PANSS) and negative symptoms a lower risk of aggressive behavior and greater improvement in medication adherence 1 year after the intervention	[188]
		Pilot study	88	No intervention	6 months	Schizophrenics with metabolic syndrome (MetS) had significantly lower predicted FVC and FEV1 than schizophrenics without MetS Significantly more MetS patients were diagnosed with restrictive pulmonary dysfunction In comparison to patients without restrictive pulmonary dysfunction, SZ patients with restrictive pulmonary dysfunction had significantly smaller waist circumferences, less physical activity, and walked less on a 6MWT	[190]
		Control group design before and after random test	64	Patients were assigned to 24 weeks of diabetes awareness and rehabilitation training (DART; *n* = 32) group or usual care plus information group (UCI; control group 32 cases). Interviews, BMI, blood pressure, fasting blood chemistry, and acceleration measurements were included. Mixed model analysis of variance was used to analyze the data. The manual intervention included 90 min of diabetes education, nutrition, and lifestyle exercise	6 months	Lowering BMI also had significant effects on triglycerides, diabetes awareness, and physical activity, but there was no significant change in fasting blood glucose or glycated hemoglobin	[222]
		Pretest and post-test intervention design	30	One hour of therapy once a week, including 10 to 15 min of exercise Participants were provided with a diet/exercise journal, a diabetes diet, a pedometer, portion control plates, measuring cups, and resistance bands Diabetes prevention education, the integrated care program consists of primary care nurse practitioners who provide primary care, care coordination, health screening, and education for people with severe MD	12 weeks	Subjects' body weight, waist circumference, hemoglobin A1C, and blood pressure were reduced, as well as their self-knowledge and Self-Care Inventory-Revised (SCI-R) scores were improved	[223]

Depressive disorder	Basic research	Zebrafish swim against the current	120	Zebrafish AE 30 min a day	10 days	After 10 consecutive days of AE, zebrafish showed less depression-like behavior and increased levels of antidepressant biomarkers (NE, 5-HIAA) As well, AE for 10 consecutive days reduced the levels of inflammatory biomarkers (IL-1, IL-4) and depression biomarkers (cortisol)	[224]
		Mouse swimming	40	During the first week, the mice acclimated to a small water depth (5 cm) for 10 min. From the second week to the fourth week, the depth and swimming time increased from 5 to 15 cm per day and from 30 min, respectively (the second week; 10 min three times), increased to 60 min (from the third week; six 10-min sessions)	4 weeks	Swimming exercise reduced depression-like behavior and decreased serum glucose and inflammatory cytokines in mice with type 2 diabetes	[225]
	Clinical research	Randomized controlled trial	42	Exercise for 45 min three times a week, starting with 25 min on a bicycle power meter at 60–70 RPM, followed by another 20 min on a cross trainer, step machine, arm geometry, treadmill, horizontal or rowing geometry	6 weeks	Compared to the usual treatment group, participants in the exercise training program showed significant improvements in cardiorespiratory fitness, waist circumference, high-density lipoprotein (HDL), cholesterol, and at least a 50% reduction in Montgomery Depression Rating Scale (MADRS) scores	[206]
		Pilot randomized controlled trial	29	Different moderate-intensity exercises (walking, zumba, Pilates, aerobics, aerobic taekwondo, and power yoga) were performed every 2 weeks for the first 12 weeks, and follow-up activities for the last 12 weeks were selected based on the participants' average exercise enjoyment rating, with the duration increasing to 150 min of moderate-intensity activity per week	6 months	Depression symptom improvement	[208]
		2 × 2 factor randomized controlled trial design	140	Each participant was assigned 150 min of moderate exercise per week with a heart rate reserve of 40% to 60%, which corresponds to a perceived motor rating of 11–13	12 weeks	Relief of depression and relief of changes in blood sugar control	[211]

BD	Clinical research	Randomized controlled trial	62	The interventions were AE or basic body awareness therapy (BBAT) compared to a single counseling and physical activity recommendation AE with higher perceived intensity intervals are trained in rehabilitation centers (e.g, cross-trainers, jump rope, and stationary bicycles) BBAT: body scan and stretch release exercise, postural stability, movement flow, and free breathing	10 weeks	Improvements in MADRS scores and cardiovascular health were observed in the exercise group	[212]
		Cycling	18	The test starts with a 5-min warm-up at 70–80 RPM with a 25 W workload and then increases the load to 75 W for men and 50 W for women for 1 min while maintaining the same speed. Next, the load increases by 25 W every 60 s until the end of the test	30 min	Serum BDNF levels were significantly higher in all BD participants compared to healthy controls	[202]
		Walk	26	Participate in the 40-m walk group, 5 days a week	4 week	15 participants reported an improvement in well-being and 10 reported no change	[226]
			—	—	—	Both dehydroepiandrosterone (DHEA) and dehydroepiandrosterone sulfate (DHEAS) were improved after exercise	—
Anxiety	Basic research	High intensity interval training	7	Run at 8 m/min, 0% tilt for 5 days, and 10 min/day	8 weeks	Improved anxiety-like behavior in rats	[227]
		Swimming	40	The mice exercised 5 days a week from 13:00–16:00 for 4 weeks During the first week, the mice acclimated to water depth (5 cm) for 10 min From the second week to the fourth week, the depth of the water gradually increases from 5 to 15 cm, and the swimming time increases from 30 (in the second week; exercise three times for 10 min each time) Increase to 60 min (from the third week; exercise 6 times for 10 min each time). The interval between each exercise is 10 min	4 weeks	Swimming exercise reversed anxiety-like symptoms in diabetic mice compared to diabetic mice that did not exercise	[215]
		Treadmill exercise	32	The rats exercised on a treadmill at moderate intensity (15 m/min; 5°; 30 min) for 5 weeks	5 weeks	Exercise training reduced anxiety-like behavior in both diabetic and nondiabetic rats	[216]
	Clinical research	Randomized controlled trial	28	The exercise schemes include the following: (1) tiptoe walking, heel walking, cross-swinging legs left and right, partial squat, single-leg squat; (2) single-leg balance, swing leg forward with knee extension, swing leg backward with knee flexion; (3) blind single-leg balance and side lunge. The duration of the exercise program was 45 min, with 1 min of rest, followed by 5 min of tr 2 months	2 months	Exercise improves functional ability, anxiety, and depression in patients with diabetic neuropathy (DN)	[218]
			53	In the experimental group, patients performed 45 to 60 min of AE training three times a week for 8 weeks, and their heart rates were maintained at 60%–70% of the ergonomic bicycle's heart rate reserve	8 weeks	AE training had significant effects on mental health, physical symptom subscales, anxiety, and insomnia	[228]
		Randomized controlled trial	49	Women with type 2 diabetes walked on a moderate-intensity treadmill at 40%–60% of their age-adjusted maximum heart rate, three times a week, on alternate days, for 12 weeks	12 weeks	Anxiety, depression, and social well-being scores improved	[219]
		Randomized controlled trial	130	Both the nondiabetic group and the diabetic group participated in hydro-high-intensity interval training (HIIT) 24 times, 48 h before and after hydro-HIIT, and quantified GDLAM index, depression, anxiety scores, and markers of oxidative dysfunction	—	After intervention, the anxiety parameters were improved in the diabetic group	[229]
		Randomized controlled trial	60	The intervention group exercised an average of 45 to 60 min three times a week for 12 weeks. The first phase starts with 45 min and works up to 60 min. The workouts begin with stretching and jogging, followed by 15 to 30 min of endurance training on a treadmill, and conclude with cooling down	12 weeks	AE training had significant effects on the subscale of physical symptoms of anxiety and insomnia in patients with type 2 diabetes	[210]
		Randomized controlled trial	24	AE, resistance exercise, combined aerobic and resistance exercise and control group, structured exercise, walking one mile, with no more than 1 day between exercises, AE group 5 days a week, with up to 50 min of exercise per day	4 months	AE alone was able to improve anxiety in patients with type 2 diabetes compared to the nonexercise group	[230]
		Randomized controlled trial	227	People were randomly assigned to either yoga or exercise. The yoga group practiced yoga under supervision for 2 weeks and alone for 3 months. The exercise group walked briskly for 30 min a day, 5 days a week	3 months	Yoga as a lifestyle is more effective than exercise alone in improving blood sugar control and anxiety	[220]

Abbreviations: AE, aerobic exercise; BDNF, brain-derived neurotrophic factor; MD, mental disorder; T2DM, type 2 diabetes mellitus.

## Data Availability

The data availability is not applicable to this review article.

## Nomenclature

T2DM: Type 2 diabetes mellitus
MDs: Mental disorders
AE: Aerobic exercise
CBT: Cognitive behavioral therapy
IR: Insulin receptor
Foxa2: Forkhead box protein A2
GLUT-4: Glucose transporter 4
PDE3B: Phosphodiesterase 3B
GSK3β: Glycogen synthase kinase 3β
LPS: Lipopolysaccharides
MGB: Microbiota–gut–brain
CNS: Central nervous system
HPA: Hypothalamic–pituitary–adrenal
CRH: Corticotrophin-releasing hormone
CRP: C-reactive protein
SNS: Sympathetic nervous system
BMI: Body mass index
PANSS: Positive and negative symptom scales
BDNF: Brain-derived neurotrophic factor
DHEA: Dehydroepiandrosterone
DHEAS: Dehydroepiandrosterone sulfate
DN: Diabetic neuropathy
IDO: Indoleamine 2,3-dioxygenase
TDO: Tryptophan 2,3-dioxygenase
PDE3B: Phosphodiesterase
IRS-1: Insulin signaling protein-1
BBB: Blood–brain barrier
GABA: Gamma-aminobutyric acid
TrkB: Tyrosine kinase receptor B
AchE: Acetylcholinesterase
PI3K: Phosphatidylinositol 3-kinase
IGF-1: Insulin-like growth factor 1
VEGFA: Vascular endothelial growth factor A
FGF-2: Fibroblast growth factor 2
HIIT: High-intensity interval training
EE: Enriched environmental
SCFAs: Short-chain fatty acids
G-CSF: Granulocyte colony-stimulating factor
MetS: Metabolic syndrome
BBAT: Basic body awareness therapy
MADRS: Montgomery Asperger's Rating Scale
VR: Virtual reality.
